# Genomic Investigation of Antimicrobial-Resistant *Salmonella enterica* Isolates From Dead Chick Embryos in China

**DOI:** 10.3389/fmicb.2021.684400

**Published:** 2021-08-23

**Authors:** Mohammed Elbediwi, Yanting Tang, Dawei Shi, Hazem Ramadan, Yaohui Xu, Sihong Xu, Yan Li, Min Yue

**Affiliations:** ^1^Department of Veterinary Medicine, Institute of Preventive Veterinary Sciences, Zhejiang University College of Animal Sciences, Hangzhou, China; ^2^National Institutes for Food and Drug Control, Beijing, China; ^3^Hygiene and Zoonoses Department, Faculty of Veterinary Medicine, Mansoura University, Mansoura, Egypt; ^4^Bacterial Epidemiology and Antimicrobial Resistance Research Unit, United States National Poultry Research Center, United States Department of Agriculture, Agricultural Research Service (USDA-ARS), Athens, GA, United States; ^5^College of Veterinary Medicine, Henan University of Animal Husbandry and Economy, Zhengzhou, China; ^6^Hainan Institute of Zhejiang University, Sanya, China; ^7^Zhejiang Provincial Key Laboratory of Preventive Veterinary Medicine, Hangzhou, China; ^8^State Key Laboratory for Diagnosis and Treatment of Infectious Diseases, National Clinical Research Center for Infectious Diseases, National Medical Center for Infectious Diseases, The First Affiliated Hospital, College of Medicine, Zhejiang University, Hangzhou, China

**Keywords:** *Salmonella*, chicken embryo, antimicrobial resistance, correlation, genomic characterization, virulence factor

## Abstract

*Salmonella* spp. is recognized as an important zoonotic pathogen. The emergence of antimicrobial resistance in *Salmonella enterica* poses a great public health concern worldwide. While the knowledge on the incidence and the characterization of different *S. enterica* serovars causing chick embryo death remains obscure in China. In this study, we obtained 45 *S. enterica* isolates from 2,139 dead chick embryo samples collected from 28 breeding chicken hatcheries in Henan province. The antimicrobial susceptibility assay was performed by the broth microdilution method and the results showed that 31/45 (68.8%) isolates were multidrug-resistant (≥3 antimicrobial classes). Besides the highest resistance rate was observed in the aminoglycoside class, all the isolates were susceptible to chloramphenicol, azithromycin, and imipenem. Furthermore, genomic characterization revealed that *S*. Enteritidis (33.33%; 15/45) was a frequent serovar that harbored a higher number of virulence factors compared to other serovars. Importantly, genes encoding β-lactamases were identified in three serovars (Thompson, Enteritidis, and Kottbus), whereas plasmid-mediated quinolone resistance genes (*qnrB4*) were detected in certain isolates of *S*. Thompson and the two *S*. Kottbus isolates. All the examined isolates harbored the typical virulence factors from *Salmonella* pathogenicity islands 1 and 2 (SPI-1 and SPI-2). Additionally, a correlation analysis between the antimicrobial resistance genes, phenotype, and plasmids was conducted among *Salmonella* isolates. It showed strong positive correlations (*r* < 0.6) between the different antimicrobial-resistant genes belonging to certain antimicrobial classes. Besides, IncF plasmid showed a strong negative correlation (*r* > −0.6) with IncHI2 and IncHI2A plasmids. Together, our study demonstrated antimicrobial-resistant *S. enterica* circulating in breeding chicken hatcheries in Henan province, highlighting the advanced approach, by using genomic characterization and statistical analysis, in conducting the routine monitoring of the emerging antimicrobial-resistant pathogens. Our findings also proposed that the day-old breeder chicks trading could be one of the potential pathways for the dissemination of multidrug-resistant *S. enterica* serovars.

## Introduction

*Salmonella* is a Gram-negative bacterium and a member of the *Enterobacteriaceae* family (Boyle et al., 2007). *S. enterica* is an imperative foodborne pathogens and is capable of causing enteric gastroenteritis (CDC, 2013; Liu et al., 2020). *S. enterica* subsp. *enterica* includes more than 2,600 serovars and can infect both humans and animals (Biswas et al., 2019; Paudyal et al., 2019). *S. enterica* is a widespread pathogen in poultry farms that can be disseminated horizontally and vertically, causing massive economic losses to the poultry industries (Bengtsson and Greko, 2014; Jajere, 2019; Xu et al., 2020b).

*S. enterica* serovars have been previously reported in many countries as a direct reason for a high number of poultry outbreaks (European Food Safety Authority and European Centre for Disease Prevention and Control, 2017; Hazards EPanelo et al., 2019; Wibisono et al., 2020). Poultry and poultry products are one of the potential pools of bacterial pathogens carrying multidrug resistance. The frequent utilization of antimicrobials in agriculture for growth promotion and bacterial infection treatment can lead to the evolution of the susceptible to antimicrobial-resistant pathogens (Crump et al., 2015; Jajere, 2019). In several reports, poultry products have been proposed as a common reservoir to multidrug-resistant (MDR) *S. enterica* serovars (Vo et al., 2006; Boyle et al., 2007; Jiang et al., 2019).

Although China is one of the key producers and consumers of poultry products across the world (USDA, 2020), still there is a knowledge gap about the frequency of *S. enterica* serovars causing chick embryo death in China (Yue et al., 2020). In this study, we investigated the prevalence of different *S. enterica* serovars isolated from dead chick embryos in Henan province, China. Additionally, we performed antimicrobial susceptibility testing to investigate the antimicrobial resistance phenotypes of the examined *S. enterica* serovars. Furthermore, genomic characterization, phylogenomic tree, and correlation analyses were conducted in order to scrutinize the potential relationship between the bacterial resistance phenotype, antimicrobial-resistant (AR) determinants, and accompanying plasmids. Here, as a part of the surveillance program, we demonstrated that routine genomic sequencing combined with an advanced analytic approach could accelerate the recognition of novel threats with significant public health concerns.

## Materials and Methods

### Sample Collection

**Figure 1:**
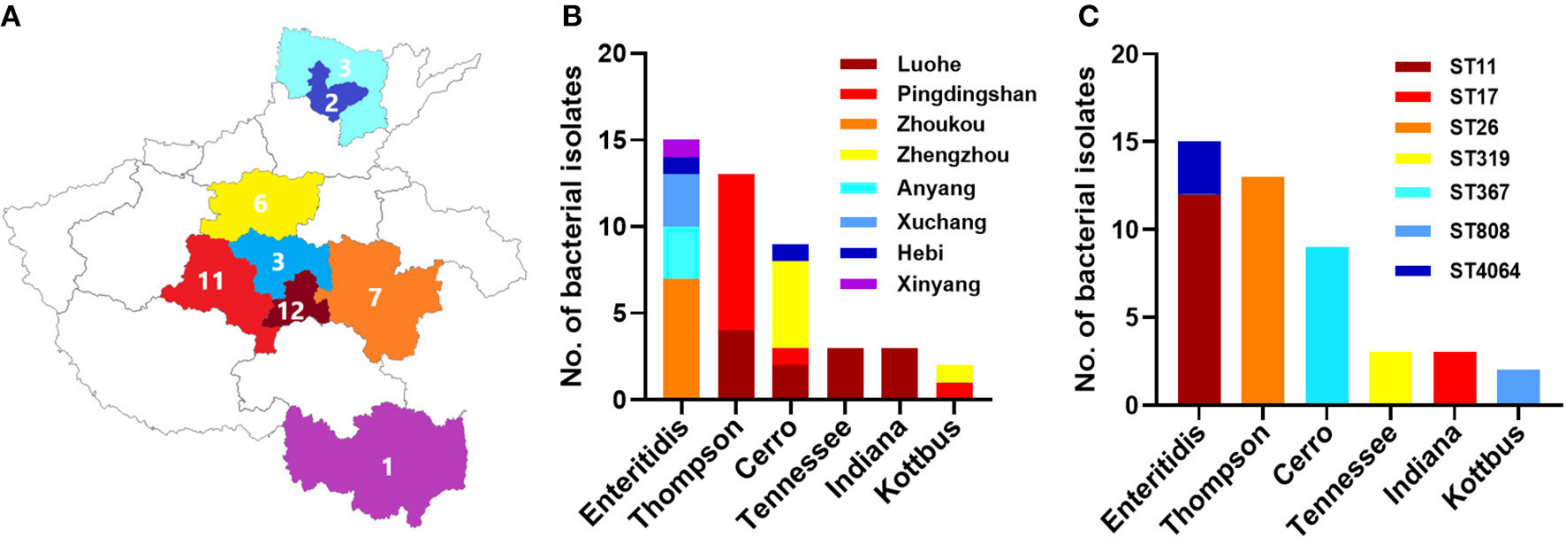
The distribution of different serovars among eight cities in Henan province, China. **(A)** The geographical distribution of the *Salmonella* isolates in Henan province with eight cities which were examined in current investigations. N.B., The number indicates the numbers of isolates collected from individual city. **(B)** The distribution of six serovars of *Salmonella* isolates. The dominant serovars are *S*. Enteritidis, *S*. Thompson, and *S*. Cerro. **(C)** The prevalence of individual serovar with their sequence type (ST) detected in this study.

The methods of sampling and isolation were described in our previous published studies (Wang et al., 2019; Xu et al., 2020b; Jiang et al., 2021). Between August 2014 and April 2015, a total of 2,139 dead chick embryo samples were collected from 28 randomly selected breeding chicken hatcheries from nine cities in Henan province: Zhengzhou, Xuchang, Pingdingshan, Hebi, Anyang, Zhoukou, Shangqiu, Xinyang, and Luohe (Figure 1A). The surface of the embryo was placed on the clean bench's sterile tray after disinfection with ethanol. Sterile forceps and scissors were used to find and extract the yolk sac of the chicken embryo samples. The yolk sac solution was enriched in 100 mL of buffered peptone water (BPW) and incubated overnight at 37°C. The solution was placed on *Salmonella*-*Shigella* agar and streaked with disposable sterile inoculating loops, and the plates were then incubated at 37°C for 24 h (Xu et al., 2020b). *Salmonella* was considered presumptive of the translucent colorless or black center colonies (Xu et al., 2020b; Jiang et al., 2021).

### *Salmonella* Isolation and Identification

In total, 45 *Salmonella* isolates were obtained among the samples. These isolates were conducted for further antimicrobial susceptibility testing and genomic characterization analysis. The distribution of the 45 selected isolates in different cities was as follows: 12 isolates from Luohe, 11 isolates from Pingdingshan, 7 isolates from Zhoukou, 6 isolates from Zhengzhou, 3 isolates from Anyang, three isolates from Xuchang, 2 isolates from Hebi, and 1 isolate from Xinyang.

### *Salmonella* Serotyping

*Salmonella* isolates serotyping was conducted by use of a classic slide agglutination assay with anti-O and anti-H serum (Tianrun Bio-Pharmaceutical, Ningbo, China). For those isolates with inconsistent results when comparing with the *in silico* prediction, we further evaluated these isolates by using serum purchased from Denmark (SSI Diagnostica, Denmark). The results were analyzed and interpreted according to the Kauffmann-White scheme, as conducted by previous publications (Pan et al., 2019).

### Antimicrobial Susceptibility Testing

The isolates were subjected to an antimicrobial susceptibility testing assay using the broth microdilution method, as described previously (Elbediwi et al., 2020b; Xu et al., 2020b; Yu et al., 2020). In total, 14 antimicrobials belonging to 10 classes were used for this assay. The cut-off values recommended by CLSI 2019 guidelines (Clinical Laboratory Standards Institute, 2019) were used for the categorization of results, and the intermediate strain, if available, was merged with the resistant strains for ease of analysis. The concentration range (μg/ml) of antimicrobials used in this assay were as follows: ampicillin (AMP): 0.5–64; amoxicillin/clavulanate potassium (AMC): 0.5/0.25–64/32; gentamicin (GEN): 0.25–32, kanamycin (KAN): 0.5–64, streptomycin (STR): 0.5–64; tetracycline (TET): 0.5–64; ciprofloxacin (CIP): 0.015–8; nalidixic (NAL): 0.5–64; chloramphenicol (CHL): 0.5–64; ceftiofur (CF): 0.015–8; cefoxitin (CX): 0.5–64; trimethoprim/sulfamethoxazole (TST): 0.06/1.19–32/608; azithromycin (AZI): 0.5–64; and imipenem (IMP): 0.015–8. We defined the isolates which could resist three antimicrobial classes or more as multidrug-resistant (MDR). The Two quality control strains, including *E. coli* ATCC 25922 and *Pseudomonas aeruginosa* ATCC 27853, were used to validate the antimicrobial susceptibility testing.

### DNA Extraction, Genome Sequencing, and Bioinformatics Analysis

The genomic DNA of the isolates was extracted and purified using QIAamp DNA mini-Kit (German Qiagen Company, Art. No. 51304), according to the manufacturer's recommended protocols. The Genomic DNA library was constructed using Nextera XT DNA library construction kit (American Illumina Company, model: FC-131-1024); then the genomic sequencing was conducted using Miseq Reagent Kit v2 300cycle kit (American Illumina Company, model: MS-102-2002). High-throughput genome sequencing was accomplished by an Illumina Miseq sequencing platform. After the sequencing results were returned, all subsequent biological information analysis was performed on the in-house Galaxy platform as described previously (Liu et al., 2021). The quality of sequencing and trimming was checked with a fast QC toolkit, while low-quality sequences and joint sequences were removed with trimmomatic (Bolger et al., 2014). The raw sequence data were under quality check and assembled by using SPAdes 4.0.1 (Bankevich et al., 2012) using the “careful correction” option to reduce the number of mismatches in the final assembly with automatically chosen k-mer values by SPAdes. The QUAST (Gurevich et al., 2013) tool was used to evaluate the assembled genomes through basic statistics generation, including the total number of contigs, contig length, and N50. After the completion of data assembly, serovar prediction was performed with SISTR v1.0.2 (Yoshida et al., 2016) and Seqsero v1.0.0 (www.denglab.info/SeqSero) by using default parameters, and MLST (https://github.com/tseemann/mlst) tools in the local Galaxy platform were used to analyze the bacterial genotype.

Furthermore, the virulence genes, antimicrobial resistance genes, and plasmid types were detected using the abricate tool (https://github.com/tseemann/abricate). This phylogenomic tree was conducted through whole-genome multilocus sequence typing (wgMLST) using the cano-wgMLST_BacCompare software that was reported recently (Liu et al., 2019) using default parameters.

### Statistical Analyses

The Pearson's correlation (*r*) between antimicrobial resistance genes, phenotypes, and plasmids among *Salmonella* isolates was measured. Resistance phenotype against the individual antimicrobial drug, antimicrobial resistance gene, and plasmid presence received scores of 1, whereas susceptibility to antimicrobials and the absence of (AR) genes or the plasmids received scores of 0. Binary data (0/1) were imported into R software (version 3.6.1; https://www.r-project.org), and correlation was determined using the “cor” function and visualized using the “corrplot” function. The significance of correlation (*P* < 0.05) was also determined using “cor.mtest” function. The correlation was considered strong if the *r* ≥ 0·6, moderate if the *r* value was between 0.4 and 0.6, and weak if *r* < 0.4 (Kirch, 2008).

## Results

### Prevalence and Geographical Distribution of *Salmonella* Serovars

A total of 45 (2.1%) *Salmonella* isolates were obtained from 2,139 chick embryo yolk samples (Supplementary Material). These 45 *Salmonella* isolates were identified in 13 out of 28 hatcheries (Supplementary Table 1). We noticed that the prevalence of *Salmonella* isolates in the hatcheries ranged between (0.8 and 9.1%), and the most contaminated hatchery was PDS2 in Pingdingshan city. The serotyping was conducted by using SISTR v1.0.2, Seqsero v1.0.0 tools, and slide agglutination assay with anti-O and anti-H serum. According to the two different *in silico* analyses, delivering identical results, we found that these 45 isolates belonged to six serovars. Additionally, the serum agglutination assay has confirmed this result. The isolates were widely distributed among Henan province cities, and the city with the highest frequent distribution was Luohe with 12 isolates followed by Pingdingshan with 11 isolates (Figure 1A). Moreover, the highest frequent serovar was *S*. Enteritidis (33.33%; 15/45) that was predominately identified in 5 out of 13 hatcheries, followed by *S*. Thompson (28.89%; 13/45), *S*. Cerro (20.00%; 9/45), *S*. Tennessee (6.67%; 3/45), *S*. Indiana (6.67%; 3/45), and *S*. Kottbus (4.44%; 2/45) (Figure 1B, Table 1).

**Table 1:** Prevalence of *Salmonella* isolates in each hatchery located in Henan province, China.

**Hatchery ID**	**City**	**Number of samples**	**Number of isolated *Salmonella***	**Prevalence**	**Serotyping (%)**
AY1	Anyang	41	3	7.317073%	*S. enteritidis* 3(100%)
HB1	Hebi	20	1	5%	S. Cerro 1 (100%)
HB3	Hebi	101	1	0.990099%	*S. Enteritidis* (100%)
LH1	Luohe	171	8	4.093567%	S. Thompson 5(62.5%) S. Cerro 2 (25%) S. Tennessee 1 (12.5%)
LH2	Luohe	120	5	4.166667%	S. Indiana 3 (60%) S. Tennessee 2 (40%)
PDS2	Pingdingshan	120	11	9.166667%	S. Thompson 9 (81 %) S. Cerro 1 (4.5%) S. Kottbus 1 (4.5%)
XC1	Xuchang	120	3	2.5%	S. Enteritidis (100%)
XY1	Xinyang	120	1	0.833333%	S. Enteritidis (100%)
ZK2	Zhoukou	120	7	5.833333%	S. Enteritidis 7 (100%)
ZZ2	Zhengzhou	60	3	5%	S. Cerro 3 (100%)
ZZ5	Zhengzhou	118	1	0.847458%	S. Kottbus 1 (100%)
ZZ6	Zhengzhou	58	1	1.724138%	S. Cerro 1 (100%)
ZZ7	Zhengzhou	60	1	1.666667%	S. Cerro 1 (100%)

### Antimicrobial Phenotype and Related Resistant Determents

**Figure 2:**
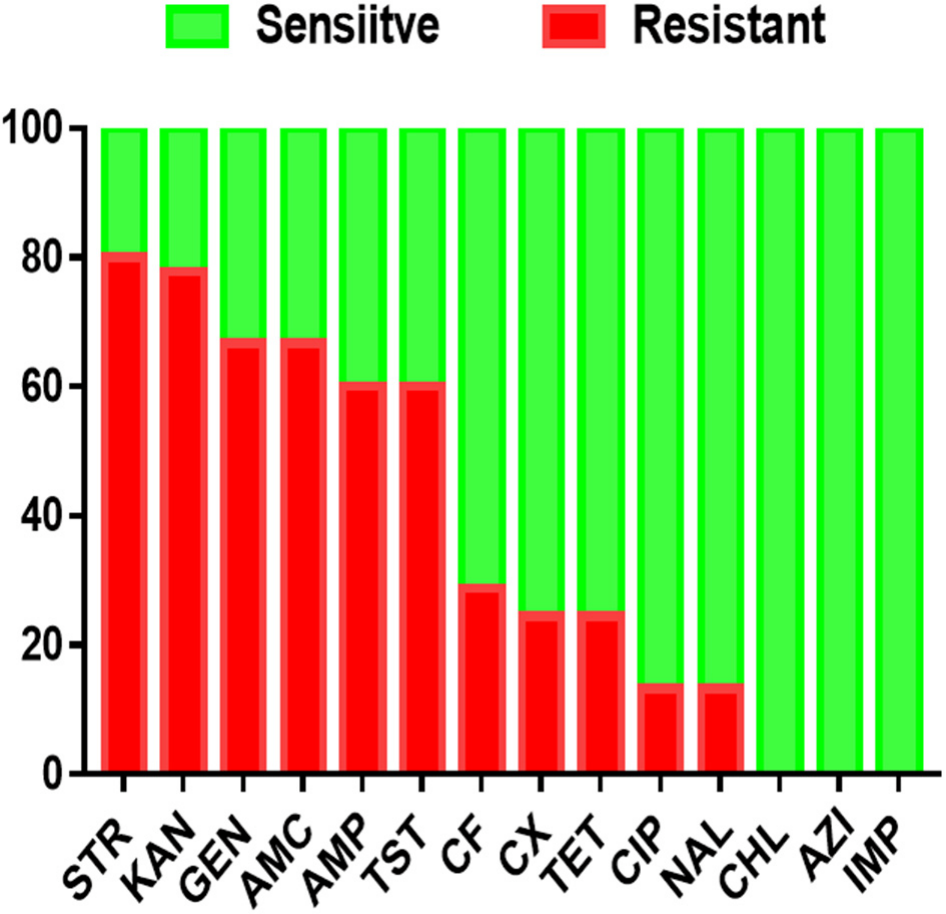
The antimicrobial resistance profile of *S. enterica* isolates was identified in this study. N.B., X-axis indicates antimicrobials and Y-axis indicates *Salmonella* isolates number.

**Figure 3:**
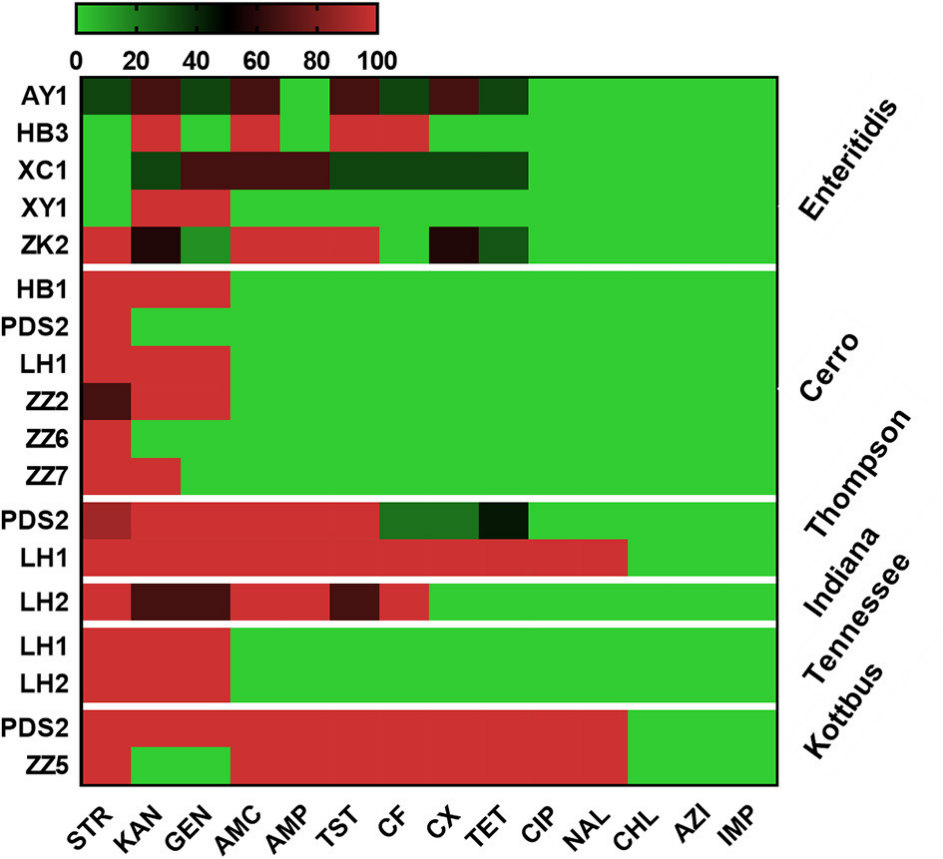
Heat map showing the antimicrobial resistance profile of different serovars isolated from different hatcheries. N.B., X-axis indicates antimicrobials and Y-axis indicates *Salmonella* serovars and Hatcheries. The antimicrobials used in this assay were as follows: ampicillin (AMP), amoxicillin and clavulanate potassium (AMC), gentamicin (GEN), kanamycin (KAN), streptomycin (STR), tetracycline (TET), ciprofloxacin (CIP), nalidixic (NAL), chloramphenicol (CHL), ceftiofur (CF), cefoxitin (CX), trimethoprim and sulfamethoxazole (TST), azithromycin (AZI), and imipenem (IMP). The abbreviations on the left side of the Y axis refer to the hatcheries located in Henan city. AY1 in Anyang city, HB1 and HB3 in Hebi city, LH1 and LH2 in Luohe city, PDS2 in Pingdingshan city, XC1 in Xuchang city, XY1 in Xinyang city, ZK2 in Zhoukou, ZZ2, ZZ5, ZZ6, and ZZ7 in Zhengzhou city. The scale showed the percentage of resistance for the isolates (from 0 to 100%). The strength of the colors corresponds to the numerical value of the prevalence of resistant isolates. Green revealed to susceptible and red revealed to resistant isolates.

Antimicrobial resistance profiles of the examined *Salmonella* isolates were determined by the broth microdilution method; accordingly, the antimicrobial resistance genes were predicted by using whole-genome sequencing data. The results obtained were classified as susceptible, intermediate resistance, and resistant. Additionally, in order to facilitate results interpretation, intermediate isolates have been considered resistant. These results showed that the higher resistance was observed in the aminoglycoside class, including STR (*n* = 36, 80%), KAN (*n* = 35, 77.7%); GEN (*n* = 30, 66.6%), while all isolates showed susceptibility for IMP, CHL and AZI (Figure 2). The antimicrobial susceptibility assay analysis showed that 68.9% (31/45) of the studied isolates were MDR. The results also revealed that *S*. Thompson obtained from the hatchery LH1 (Luohe city) and *S*. Kottbus obtained from the hatchery PDS2 (Pingdingshan city) had the highest resistance rate among all serovars, showing a resistance profile toward 11 antimicrobial drugs (STR, KAN, GEN, AMC, AMP, TST, CF, CX, TET, CIP, and NAL) (Figure 3). *S*. Cerro isolates were observed to be resistant against the aminoglycoside class (Figure 3).

**Figure 4:**
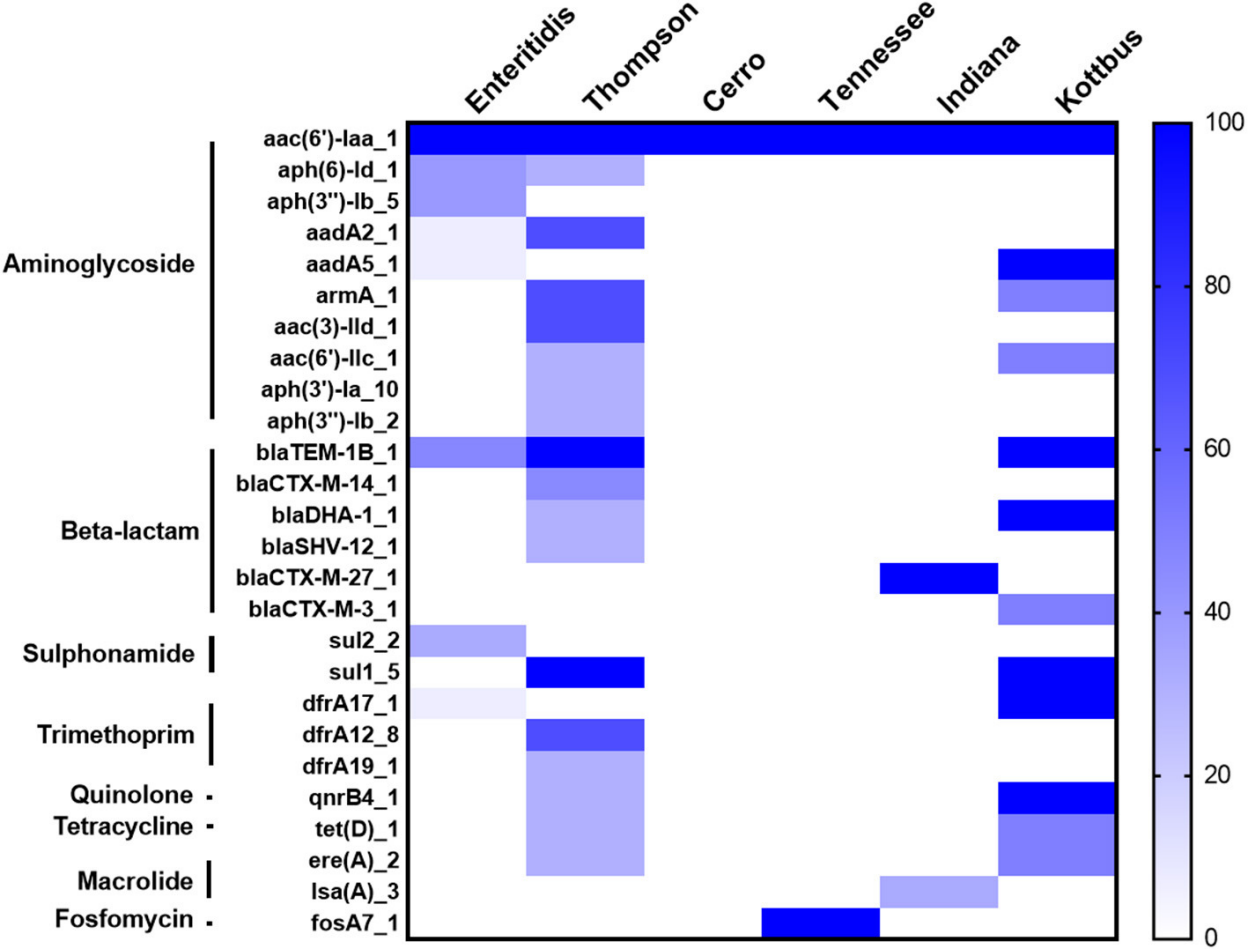
The heatmap of antimicrobial resistance (AR) genes in the recovered *S. enterica* isolates. All the studied isolates harbored *aac(6*′*)-Iaa* gene encoding resistance to aminoglycoside, and *S*. Cerro isolates only carried it. The scale showed the prevalence of AR genes among the isolates (from 0 to 100%), The strength of the colors corresponds to the numerical value of AR genes prevalence. Dark blue color revealed to high prevalence and white color revealed gene absence.

Furthermore, the screening for the resistance determinants in the whole genomic sequence (WGS) of these isolates was in concordance with the phenotypic resistance. WGS analysis showed that all the examined isolates harbored the *aac(6*′*)-Iaa* gene, conferring resistance to aminoglycoside (Figure 4). Importantly, plasmid-mediated quinolone resistance genes (*qnrB4*) were detected in certain *S*. Thompson and all *S*. Kottbus isolates. Genes encoding β-lactamases were identified in three serovars (*S*. Thompson, *S*. Enteritidis, *S*. Kottbus). The gene *sul1*, which encodes resistance to sulfonamide, was only found in *S*. Thompson and *S*. Kottbus, while *Sul2* was only detected in *S*. Enteritidis serovar. Regarding serovars distribution, our results founded that the prevalence of antimicrobial resistance genes in *S*. Thompson was the highest compared to the other serovars.

### Whole Genome Sequencing and Bioinformatics Analysis

After conducting the whole genome sequencing and genomic assembly of the *Salmonella* isolates, the number of contigs was calculated to be between 22 and 100 contigs. Genome sequencing and assembly results parameters are summarized in Table 2. The average genome size of draft assemblies was 4,917,882 bp. Furthermore, the average N50 was 398,064 bp. With further WGS analysis, we found that MLST patterns were assigned based on the allelic profile of each isolate, and the results showed that the *Salmonella* isolates belonged to seven Sequence Types (STs) (Figure 1C). More than half of the isolates (55.56%) were assigned to ST26 (28.9%) and ST11 (26.7%). Our results also showed that 80% of *S*. Enteritidis isolates belonged to ST11 and 20% belonged to ST4064. All *S*. Thompson isolates belonged to ST26, and *S*. Tennessee, *S*. Indiana, and *S*. Kottbus belonged to ST319, ST17, ST808, respectively.

**Table 2:** The quality assessment for all the assembled genomes examined in this study.

**Assembly**	**# Contigs (≥0 bp)**	**# Contigs (≥1,000 bp)**	**Total length (≥0 bp)**	**Total length (≥1,000 bp)**	**# Contigs**	**Largest contig**	**Total length**	**GC (%)**	**N50**	**N75**	**L50**	**L75**	**# N's per 100 kbp**
SAL03382	179	34	5,101,129	5,069,248	43	1,459,447	5,075,471	51.89	549,762	206,787	3	8	13.5
SAL03381	174	32	5,122,141	5,093,922	42	1,129,176	5,100,360	51.91	427,932	185,546	4	10	9.29
SAL03380	170	21	5,045,692	5,012,793	29	1,459,263	5,017,756	51.88	554,412	226,286	3	6	9.33
SAL03379	81	21	4,731,474	4,719,477	22	1,549,532	4,720,231	52.1	478,743	305,471	3	6	8.37
SAL03378	102	24	4,781,062	4,762,535	25	1,550,631	4,763,192	52.08	478,625	229,238	3	6	10.18
SAL03377	162	22	4,753,471	4,718,123	25	1,550,267	4,719,884	52.1	479,674	229,550	3	6	10.17
SAL03376	235	22	4,804,954	4,750,264	24	1,550,631	4,751,718	52.09	478,743	228,572	3	6	6.06
SAL03375	102	23	4,771,947	4,752,092	26	1,550,531	4,754,283	52.09	479,247	183,592	3	7	8.58
SAL03374	83	22	4,758,006	4,743,809	24	1,550,333	4,745,119	52.08	479,038	305,478	3	6	7.99
SAL03373	1,408	20	5,158,260	4,716,945	100	1,550,272	4,765,377	51.97	478,743	305,792	3	6	6.55
SAL03372	99	22	4,712,220	4,693,180	25	1,550,242	4,695,440	52.14	478,743	229,451	3	6	12.35
SAL03371	246	49	4,869,844	4,820,555	59	491,585	4,827,171	51.91	163,089	118,349	10	19	3.94
SAL03370	466	218	5,625,106	5,553,117	260	404,605	5,583,093	51.42	96,096	53,751	17	35	16.44
SAL03369	197	30	5,126,187	5094110	37	1,280,533	5,099,320	51.91	427,931	185,546	4	9	5.65
SAL03368	174	28	5,024,981	4,995,172	38	1,431,046	5,001,610	51.95	427,931	217,607	4	8	7.82
SAL03367	161	29	5,210,710	5,184,301	38	1,431,045	5,190,098	51.84	427,931	217,607	4	8	5.51
SAL03366	81	22	4,706,754	4,692,495	24	1,550,142	4,693,910	52.13	479,247	228,503	3	6	8.37
SAL03365	85	21	4,706,547	4,691,715	23	1,551,525	4,693,016	52.14	478,531	305,799	3	6	6.16
SAL03364	106	21	4,712,417	4,690,024	28	1,550,340	4,694,627	52.14	478,743	305,799	3	6	4.11
SAL03363	79	22	4,705,365	4,691,095	26	1,550,240	4,694,110	52.14	478,531	305,799	3	6	8.35
SAL03362	89	20	4,711,773	4,695,761	22	1,549,498	4,697,071	52.14	479,247	227,733	3	6	6.22
SAL03361	157	20	4,728,930	4,694,640	22	1,549,402	4,696,094	52.14	489,575	227,891	3	6	10.18
SAL03360	107	22	4,713,218	4,692,254	24	1,550,142	4,693,708	52.14	479,037	217,535	3	6	6.24
SAL03359	171	36	5,213,277	5,186,643	46	824,144	5,193,128	51.84	427,931	178,887	5	11	1.85
SAL03358	159	27	5,021,552	4,995,614	35	1,430,953	5,000,796	51.95	512,610	227,901	3	7	3.8
SAL03357	350	30	5,069,148	4,991,023	44	1,280,532	4,999,671	51.95	330,177	185,546	4	10	3.84
SAL03356	180	30	5,219,669	5187,707	39	1,431,046	5,193,505	51.84	427,931	238,251	4	7	7.41
SAL03355	208	43	5,162,509	5,128,258	55	750,697	5,136,573	51.92	427,931	191,899	5	9	5.72
SAL03354	199	44	5,159,850	5,129,202	54	750,567	5,136,673	51.92	513,033	191,899	4	9	5.68
SAL03353	202	43	5,160,755	5,128,194	54	1,330,835	5,136,006	51.92	330,277	191,899	4	9	5.67
SAL03352	199	45	5,159,795	5,127,779	56	726,995	5,135,592	51.92	330,177	238,251	6	10	5.57
SAL03351	191	43	4,922,045	4,889,897	52	678,126	4,895,060	52.17	212,889	164,537	7	14	11.83
SAL03350	193	43	4,921,119	4,889,386	52	772,058	4,894,893	52.18	212,621	164,419	7	13	9.81
SAL03349	192	42	4,921,306	4,888,841	51	772,055	4,894,424	52.17	212,621	165,335	7	13	11.99
SAL03348	182	48	4,853,874	4,822,278	58	491,585	4,828,987	51.91	163,089	118,349	9	18	8.03
SAL03347	2340	310	7,989,568	7,7107,85	353	319,335	7,740,624	46.41	56,757	27,806	42	90	91.9
SAL03346	135	26	4,612,643	4,590,676	31	551,545	4,593,945	52.28	418,729	200,975	5	9	8.47
SAL03345	152	27	4,618,512	4,593,259	31	696,184	4,595,721	52.28	389,815	314,628	5	8	4.29
SAL03344	128	28	4,615,565	4,595,105	33	695,786	4,598,374	52.29	389,013	300,606	5	8	10.61
SAL03343	144	29	4,614,770	4,591,004	34	509,159	4,594,273	52.29	373,423	200,975	6	10	6.38
SAL03342	143	29	4,614,296	4,591,142	34	533,428	4,594,411	52.29	368,293	241,262	6	9	8.36
SAL03341	159	31	4,655,576	4,630,294	35	533,423	4,632,756	52.25	368,294	200,975	6	10	10.43
SAL03340	167	30	4,615,758	4,585,694	36	501,135	4,589,337	52.28	368,230	241,262	6	9	10.55
SAL03339	134	25	4,608,711	4,586,707	30	551,545	4,589,873	52.29	390,375	314,629	5	8	8.32
SAL03338	149	35	4,677,138	4,654,275	40	542,400	4,657,409	52.16	419,125	352,009	5	8	8.35
Average	240.44	40.2	4,955,324.98	4,911,586.4	49.7556	1,089,199.1	4,917,882	51.92	398,064.3	219,999.6	5.56	10.96	9.783

**Figure 5:**
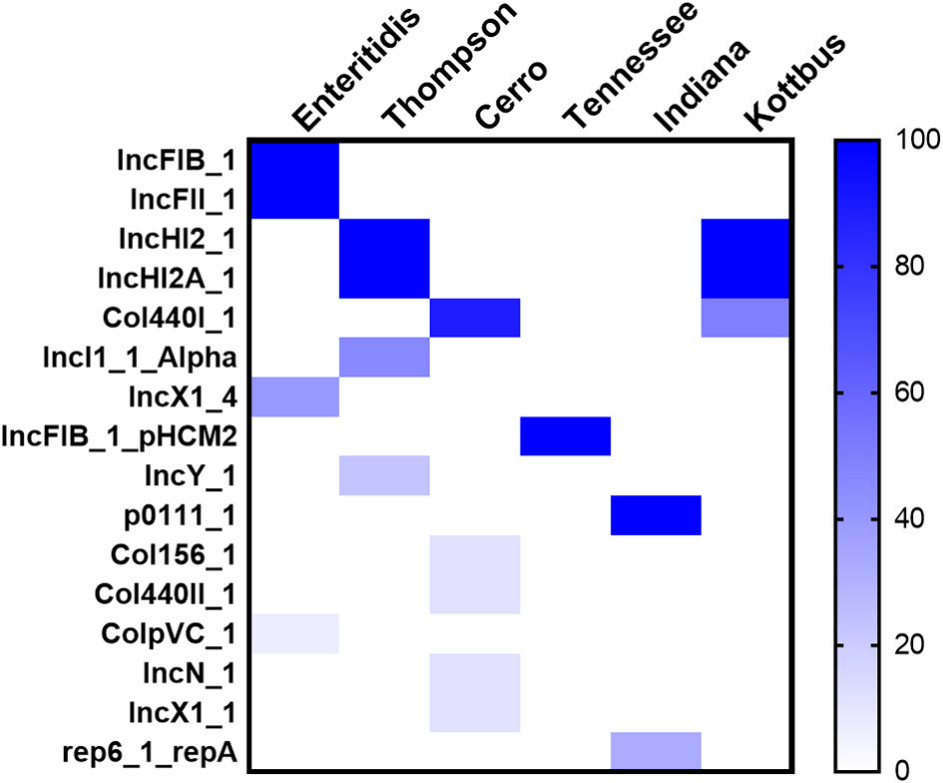
The heatmap of plasmids distribution in different *S. enterica* isolates. The scale showed the prevalence of the plasmids among the isolates (from 0 to 100%). The strength of the colors corresponds to the numerical value of the prevalence of the plasmids. Dark blue color indicates high prevalence and white color for gene absence.

Moreover, WGS also showed that the detection of 16 different plasmid replicons in the recovered *Salmonella* strains. Among the whole data, there were few plasmids carried by all strains, and the characteristics of plasmids carried by strains with different serovars were obvious. The largest number of plasmid replicons with different types have been detected in *S*. Cerro (five different plasmids), followed by *S*. Thompson and *S*. Enteritidis (four different plasmids). However, the major plasmids found in *S*. Enteritidis were IncFIB(S)_1 and IncFII(S)_1, whereas, in *S*. Thompson and *S*. Kottbus, they were IncHI2 and IncHI2A; *S*. Cerro, *S*. Tennessee, and *S*. Indiana carried the only detected plasmids Col440I_1, IncFIB(pHCM2)1_pHCM2, and p0111_1, respectively (Figure 5).

**Figure 6:**
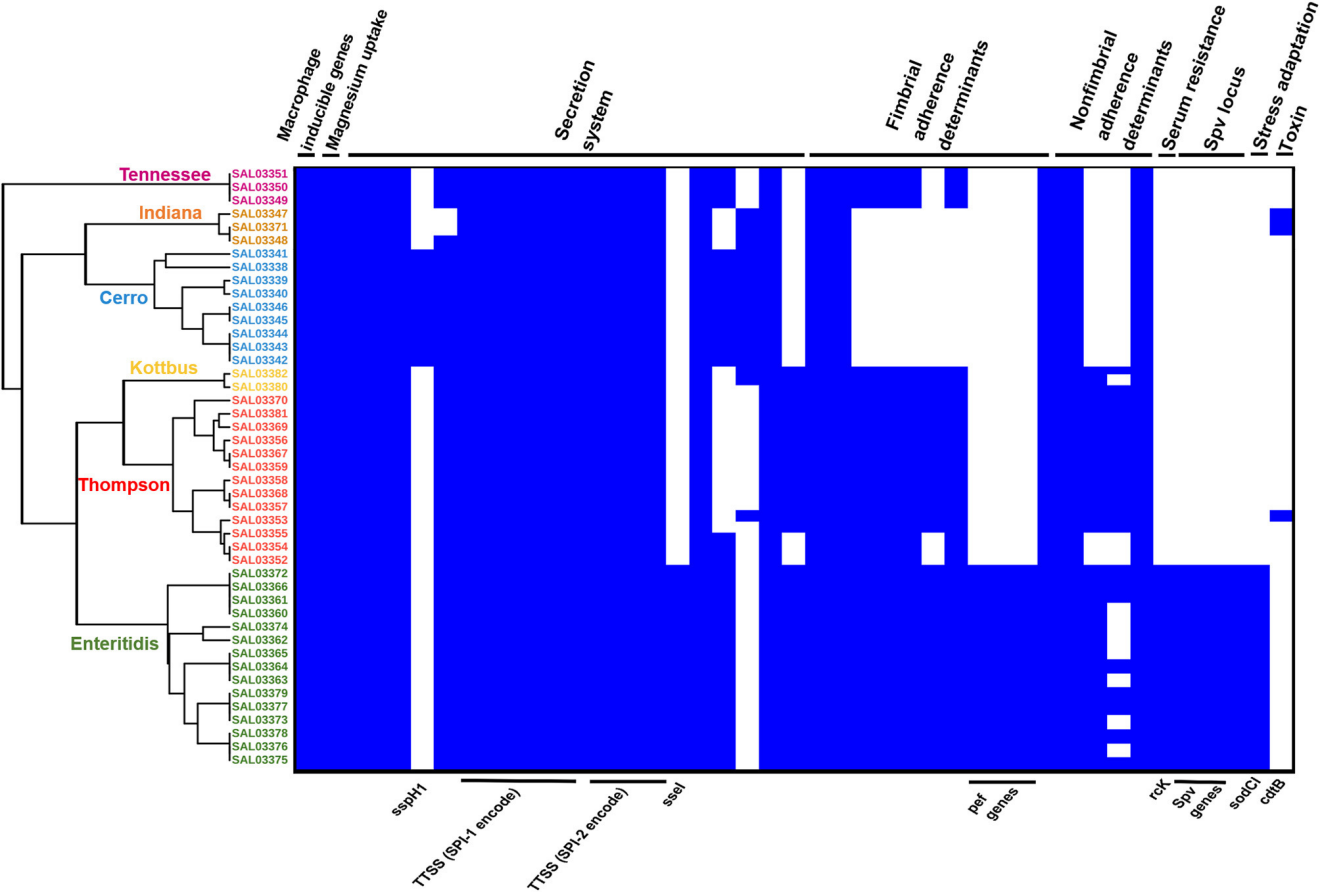
The combinatorial graph of the wgMLST phylogenomic evolutionary tree and virulence genes in the *S. enterica* isolates. The figure showed that *S. enteritidis* harbored a higher number of virulence genes compared with other serovars. In the heat map of the virulence genes, blue color refers to “presence,” white color refers to “absence.” The figure showed that *pef, rck, sodCl*, and *spv* loci in addition to *ssel* genes were detected only in the *S. enteritidis* isolates. Besides, one *S*. thompson isolate and two *S*. Indiana isolates harbored the gene *cdtB* encoding typhoid toxins. All the isolates harbored *Salmonella* pathogenicity islands 1 and 2 (SPI-1 and SPI-2) virulence factors. N.B., According to the phylogenomic tree, green color refers to *S*. Enteritidis isolates, orange color refers to *S*. Cerro isolates, brown color refers to *S*. Kottbus isolates, gray color refers to *S*. Indiana isolates, and red color refers to *S*. Thompson isolates.

Prediction of virulence genes was performed on the basis of the virulence factors database (VFDB) by using the abricate web tool, and the results are presented in Supplementary Table 2. In total, 111 virulence genes implicated in different mechanisms of virulence and pathogenicity were detected (Figure 6). Our results showed that *S*. Enteritidis harbored a higher number of virulence genes compared to other serovars. Fimbrial adherence factor Pef, serum resistance gene *rck*, stress adaptation gene *sodCl*, and the plasmid-borne *spv* locus in addition to the *ssel* gene, which plays a role in the type three secretion system, were detected only in *S*. Enteritidis isolates (Figure 6, Supplementary Table 2). We also noticed *sspH1* gene has been only detected in *S*. Cerro isolates. Importantly, we also found that one *S*. Thompson isolate (SAL03370) and two *S*. Indiana (SAL03347, SAL03348) isolates harbored the gene *cdtB* encoding typhoid toxin. Interestingly, *pltA* and *pltB* genes, which are essential for producing cytolethal distending toxin (CDT), were not found. All the isolates harbored *Salmonella* pathogenicity island 1 and 2 (SPI-1 and SPI-2) virulence factors (Figure 6).

To further investigate the genomic relationship among the isolates, we performed a phylogenomic analysis. Importantly, wgMLST analysis (Figure 6) revealed that the isolates belonging to the same serotypes are very close to each other, clustering in the same sub-clade. The phylogenomic tree suggested a strong association between *S*. Indiana and *S*. Cerro isolates and between *S*. Thompson and *S*. Kottbus isolates, which were grouped in the same sub-clade with a very similar pattern for virulence factor cassettes (Figure 6).

### Association of Phenotypic Resistance, Resistance Associated Gene, and Plasmid Among the *Salmonella* Isolates

**Figure 7:**
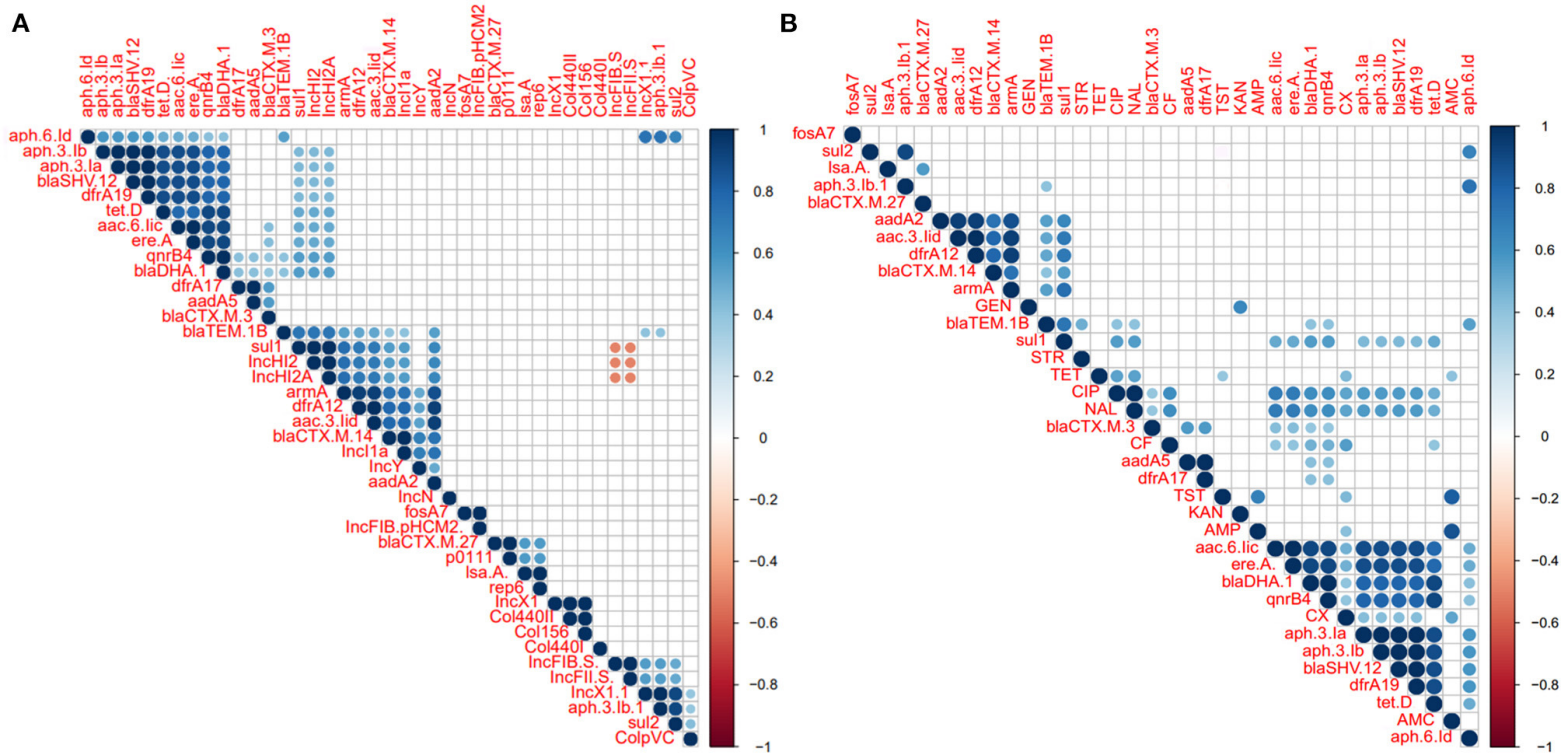
Correlation analysis between antimicrobial resistance (AR) phenotypes, AR determinants, and plasmids among the *S. enterica* isolates. **(A)** Correlation analysis between plasmids and AR determinants. **(B)** Correlation analysis between AR phenotypes and AR determinants. The blue and orange colors of boxes indicate positive and negative correlation, respectively. The strength of the colors corresponds to the numerical value of the correlation coefficient (*r*). All correlation results were statistically significant (*p* < 0.05). The results showed positive correlations (*r* < 0.6) between the different AR genes belonging to different antimicrobial classes such as those *apha.3.lb, apha.3.la, aac.6.lic, bla*_SHV−12_, and *bla*_DHA−1_, *tetD*, and *qnrB*. We also noticed that these genes showed a week correlation with IncHI2/A plasmid types and *sul1*. Additionally, IncHI2/A plasmid types showed a strong positive correlation with *sul1, dfrA12, armA*, and *bla*_TEM.1B_ genes. Interestingly, IncF plasmid showed a strong negative correlation (*r* > −0.6) with IncHI2/A plasmid.

Correlation analysis of antimicrobial resistance phenotype, antimicrobial resistance gene, and plasmid among the examined *Salmonella* isolates was performed. All observed correlation results were statistically significant (*p* < 0.05) as indicated in Figure 7. The results showed strong positive correlations (*r* < 0.6) between antimicrobial-resistant genes which belonging to different antimicrobial classes such as those remarked for aminoglycosides resistant genes (*apha.3.lb, apha.3.la, aac.6.lic), bla* genes (*bla*_SHV−12_ and *bla*_DHA−1_), *tetD*, and *qnrB* (Figure 7A). We also noticed that these genes showed a week correlation with IncHI2/A plasmid types and *sul1*. Additionally, IncHI2 and IncHI2A plasmids showed a strong positive correlation with *sul1, dfrA12, armA, and bla*_TEM.1B_ genes. *apha.6.ld* also showed strong positive correlations with *apha.3.lb, sul1*, and IncX1 (Figure 7A). Interestingly, the IncF plasmid was suggested to display a strong negative correlation (*r* > −0.6) with IncHI2 and IncHI2A plasmids (Figure 7A). Figure 7B showed the correlation analysis between the antimicrobial resistance phenotypes and antimicrobial-resistant genes. Interestingly, we found that CIP and NAL showed a strong positive correlation with each other. Besides, both of them showed a strong positive correlation with *aac.6.lic, ereA, bla*_DHA_, and *qnrB4* genes. AMP was found to be a strong positive correlation with AMC and KAN showed a strong positive correlation with GEN (Figure 7B).

## Discussion

Recently, A meta-analysis study displayed that *Salmonella* is one of the most widespread pathogenic foodborne bacteria in Chinese food products (Paudyal et al., 2018). Avian salmonellosis is listed as one of the 16 priority animal diseases according to the national medium- and long-term plan for prevention and control of animal diseases in China (2012–2020). Several studies reported that avian salmonellosis can be disseminated through fertilized eggs, leading to the chicken embryo death (EFSA, 2007; Gantois et al., 2009; Zhao et al., 2021). However, several previous studies have reported a high prevalence of *Salmonella* Gallinarum from poultry and poultry products in China. In this study, we focus on Non-Gallinarum serovars (Xu et al., 2020b). Accordingly, to better describe the actual disease burden in breeders caused by *S. enterica*, we aimed to pick up the dead embryos instead of the normal embryo samples.

Vertical transmission or transovarian transmission is one of the key routes of *Salmonella* transmission to the egg. It occurs when the egg content is contaminated with *Salmonella* during the egg formation (Messens et al., 2005). Vertical transmission is common in host restricted *Salmonella* serovars, such as *S*. Gallinarum and *S*. Pullorum, but has also been reported in typical foodborne pathogen including *S*. Enteritidis (Poppe et al., 1998). Transmission via this route is directly related to the affinity of certain serovars in the reproductive tract of the hens (EFSA, 2010). Several studies reported that *S*. Enteritidis is the predominant serovar to colonize the reproductive organs of mature laying hens and cause severe outbreaks for humans due to egg contamination across the world (Okamura et al., 2001; Gantois et al., 2009). In this study, 45 (2.1%) *S. enterica* isolates were identified from yolk samples. These isolates were subjected to antimicrobial susceptibility assay and further genomic characterization. The positive rate found in this study was lower than that observed in eggs collected from poultry farms in Yangling, Shaanxi province (6.6%) (Li et al., 2020a), and Shandong province (Zhao et al., 2021) and higher than that obtained by Li et al. (0.5%) across China in 2016 (Li et al., 2020b).

The serovars identified in this study suggested a wide range of *Salmonella* diversified serovars within chick embryos. *S*. Enteritidis is a common serovar of non-typhoid *Salmonella*, and is also an invasive pathogenic foodborne pathogen. Our results showed that *S*. Enteritidis is the dominant serovar, which is consistent with those found in breeder chick farms (Fei et al., 2018). Additionally, Li et al. reported that *S*. Enteritidis was the dominant serovar isolated from diseased or dead chicken samples in Jiangsu, Henan, Heilongjiang, and Shandong provinces in 2012 (Li et al., 2018). *S*. Enteritidis was also found to be the cause of numerous foodborne outbreaks in Henan province, China (Xia et al., 2009). Globally, several studies also reported that *S*. Enteritidis was the dominant serovar in diseased or dead chicken from Uruguay (Betancor et al., 2010), the United States (Schutze et al., 1996), India (Suresh et al., 2006), Japan (Sasaki et al., 2011), Algeria (Ayachi et al., 2015), and Canada (Poppe, 1994). Our results were somewhat contradicted with those obtained from Shandong province, reporting the dominance of *S*. Thompson in dead-in-shell chicken embryos (Zhao et al., 2021). While *S*. Indiana was found to be dominant in Shandong (Lu et al., 2011), and *S*. Weltevreden was the dominant serovar in poultry farms in Vietnam (Lettini et al., 2016). Of the remaining isolates, *S*. Thompson, *S*. Indiana, *S*. Kottbus, *S*. Tennessee, and *S*. Cerro were also commonly recovered from poultry-based products and humans in China (Bai et al., 2015; Chen et al., 2020; Yang et al., 2020). These reports provide strong evidence about the increasing prevalence of *S*. Enteritidis in the poultry farms as well as the poultry supply chain in China. Some literature proposed that the decreasing prevalence of *S*. Gallinarum and *S*. Pullorum in the global poultry industry may have allowed *S*. Enteritidis to disseminate in poultry flocks (Rabsch et al., 2001). Collectively, the epidemic severity of *S*. Enteritidis in China's chicken industry chain is of great concern.

Moreover, all isolates showed high drug resistance, of these, 68% (31/45) were multidrug-resistant, and the frequency of the aminoglycoside resistance was the highest. All strains harbored the *aac(6*′*)-Iaa* gene, which conferring aminoglycoside resistance. This inspection can be clarified by the misuse and/or overuse of antibiotics in the agriculture and veterinary sectors, where antibiotics are applied for therapeutic practices, prophylactic, and growth improvement purposes. This study also showed that the examined *Salmonella* serovars had significant behavior differences against a range of antimicrobials. Most of our examined *Salmonella* isolates exhibited resistance to three or more antimicrobials. The surge prevalence of antimicrobial-resistant *Salmonella* isolates is recognized as a crucial public health issue. The antimicrobial resistance of *S*. Thompson and *S*. Kottbus was alarming and showed high antimicrobial resistance rates among all isolates, with a widespread antimicrobial resistance spectrum. This type of bacterial resistance has been previously detected in *S*. Thompson obtained from eggs in Shaanxi province, China (Li et al., 2020a).

Although they are not the dominant serovars, multiple antimicrobial selection pressure can play an important role in converting the serovar prevalence to dominant serovars over time by increasing their virulence (Boyle et al., 2007). In contrast, the overall resistance rate of *S*. Cerro is low, presenting only resistance to the aminoglycoside drugs. These results were in agreement with several worldwide reports on the dissemination of MDR among the *Salmonella* isolates (Li et al., 2013; Abdeen et al., 2018; Chuah et al., 2018; Elbediwi et al., 2021).

Genomic analysis of *Salmonella* isolates showed the detection of different antimicrobial resistance genes, which could correlate with the high level of antimicrobial resistance. All strains harbored the *aac(6*′*)-Iaa* gene, which was believed to be responsible for aminoglycoside resistance. Additionally, other aminoglycoside resistance genes were also detected in the present study (Figure 3). Among different mechanisms of aminoglycoside resistance, enzymatic modification is the most prevalent in pathogenic bacteria, including *Salmonella* spp. (Ramirez and Tolmasky, 2010; Biswas et al., 2019). Resistance to β-lactam antimicrobials is controlled by *bla* genes, and these genes are generally hydrolyzing the β-lactam ring, leading to antibiotic inactivation. *bla* resistance genes were found in ampicillin-resistant *S*. Enteritidis, *S*. Thompson, and *S*. Kottbus isolates and one *S*. Indiana isolate in this study (Figure 5). The dominant *bla* gene conferring ampicillin resistance in most of the *Salmonella* serovars was found to carry a few different types of *bla*_TEM_ (de Toro et al., 2011; Eguale et al., 2017; García et al., 2018). TEM-2, SHV, and OXA-1 (β -lactamases) are also widespread in *Enterobacteriaceae*, although they are much rarer than TEM-1 (Livermore, 1995). We also found *sul1* and *sul2* antibiotic resistance genes in addition to other important gene *dfrA12-17-19* in trimethoprim-sulfamethoxazole-resistant isolates. It has been reported in several studies that resistance to sulphonamide antibiotics is mainly mediated by the *sul1, sul2*, and *sul3* genes (Antunes et al., 2007; Wang et al., 2014). Besides, the main mechanism of trimethoprim resistance, which is mainly mediated by *dfrA* genes, is the existence of integron-borne dihydrofolate reductases (Miko et al., 2005; Krauland et al., 2010; El-Sharkawy et al., 2017; Wang et al., 2017). Resistance to tetracyclines and quinolones antibiotics, which was conferred due to the presence of the *tetD* gene and the plasmid-mediated quinolones-resistant gene *qnrB4*, was also identified in *S*. Thompson and *S*. Kottbus serovars, posing a great public health concern. While both of them are considered critical antimicrobials, quinolones are currently preferred as a front-line drug of choice for the treatment of salmonellosis.

The genomic analysis also demonstrated that the high resistant serovars (Thompson and Kottbus) harbored IncHI2 and IncHI2A plasmids, as well as the hypervirulent serovar Enteritidis harbored IncF plasmid. Interestingly, these two plasmids were reported to be the predominant types in the MDR and hypervirulent isolates (Chen et al., 2016; Elbediwi et al., 2019, 2020a). IncHI2, IncHI2A, and IncF plasmids are often linked with persistence, as well as harboring virulence and antimicrobial resistance genes, which play a vital role in contributing to bacterial fitness (Ibarra and Steele-Mortimer, 2009; Villa et al., 2010; Coelho et al., 2012; Chen et al., 2016; Biswas et al., 2020; Xu et al., 2020a).

Considering the virulence factors that were investigated, *S*. Enteritidis isolates displayed a broader range of pathogenicity determinants as compared to other serovars. *S*. Enteritidis isolates were found to be harboring *pef* fimbrial genes, *rck* gene*, spv* locus, and *sodCl* gene. Serum resistance gene *rck* is encoding an outer membrane protein, which enhances the bacterial adhesion and invasion, also confers high resistance to the bactericidal activity of complement, by hindering the polymerization of complement component in the outer bacterial membrane (Guiney et al., 1995; Rosselin et al., 2010). Fimbrial adherence Pef and *spv* locus were previously reported as plasmid-borne determinants and have an important role in the *Salmonella* adhesion to the host gut epithelium (Guiney et al., 1995; Ledeboer et al., 2006; Yue et al., 2012, 2015). Stress adaptation gene *sodCl* is known to be involved in the *Salmonella* protection from phagocytic superoxide during infection (Sly et al., 2002; Uzzau et al., 2002). Regarding the *sspH1* gene, which has been only identified in *S*. Cerro isolates, this gene was found to play an essential role in bacterial invasion of the epithelial cells and stimulation of the intestinal inflammatory response (Haraga and Miller, 2003). Otherwise, all the examined isolates harbored the typical virulence factors from *Salmonella* pathogenicity islands 1 and 2 (SPI-1 and SPI-2). SPI-1/-2 encodes a type three secretion system (T3SS), which is important for the injection of effector proteins into the host cells for modulation of the course of *Salmonella* infection (Que et al., 2013). SPI-1 genes are responsible for the invasion of the host cells, and the host immune response regulation. Besides, it encodes transcription factors that regulate the expression of some virulence factors (Raffatellu et al., 2005; Lou et al., 2019). Moreover, the development of the systemic disease is dependent on a type III secretion system (T3SS) encoded by SPI-2. SPI-2 harboring genes that are essential for bacterial intracellular survival (Hensel, 2000; Figueira et al., 2013).

The correlation analysis displayed strong positive associations between the different antimicrobial-resistant genes, exhibiting phenotypic resistance to different antimicrobial classes, such as those remarked for aminoglycosides resistant genes*, bla* genes, *tetD*, and *qnrB*, suggesting selective pressure exerted by the misusage of these antimicrobials in poultry production (Xu et al., 2020b). Additionally, the co-occurrence of these antimicrobial-resistant genes together either on the chromosome (same gene cassette) or plasmids, could be also another potential reason for this strong relation (Wales and Davies, 2015). The analysis also showed a strong positive correlation between different antimicrobial-resistant genes and IncHI2, IncHI2A, and IncF plasmids, confirming the vital role of these plasmids in antimicrobial-resistant genes dissemination, as mentioned earlier (Elbediwi et al., 2019).

Collectively, we found a considerable diversity of *Salmonella* serovars circulation in dead chick embryos in Henan, China, with multi-drug resistance potentials. Therefore, it is essential to continue monitoring the *Salmonella* serovars and implement the relevant strategic plans for prevention and control. Systematically and continuously monitoring the antimicrobial resistance of *Salmonella*, and application of an antimicrobial management plan for rational uses of essential antimicrobials in chicken farms are essential to improve food safety and prevent the MDR bacteria emergence. Direct whole-genome sequencing surveillance could provide a more comprehensive understanding of *Salmonella* population diversity, which might project public health awareness in the course of the next epidemics.

## Data Availability Statement

The datasets presented in this study can be found in online repositories. The names of the repository/repositories and accession number(s) can be found at: https://www.ncbi.nlm.nih.gov/bioproject/PRJNA727934, PRJNA727934.

## Author Contributions

ME and YT analyzed the data and drafted the manuscript. YT did the experiments and finalized the figures. HR did the statistical analysis work and review the paper. DS, SX, YX, and YL provided essential comments and helped with the edition of the manuscript. MY conceived the idea and assisted with data analysis and writing. All authors read, revised, and approved the final manuscript.

## Conflict of Interest

The authors declare that the research was conducted in the absence of any commercial or financial relationships that could be construed as a potential conflict of interest.

## Publisher's Note

All claims expressed in this article are solely those of the authors and do not necessarily represent those of their affiliated organizations, or those of the publisher, the editors and the reviewers. Any product that may be evaluated in this article, or claim that may be made by its manufacturer, is not guaranteed or endorsed by the publisher.

## Supplementary Material

Supplementary Table 1 *Salmonella* isolates serotyping by sera agglutination assay and *in silico* analysis by using Seqsero v1.0.1 and SISTR v1.0.0.

Supplementary Table 2 The virulence factor profiling for all the examined *Salmonella* isolates in this study.
